# From Childhood Interpersonal Trauma to Binge Eating in Adults: Unraveling the Role of Personality and Maladaptive Regulation

**DOI:** 10.3390/nu16244427

**Published:** 2024-12-23

**Authors:** Lily Bellehumeur-Béchamp, Maxime Legendre, Catherine Bégin

**Affiliations:** 1 School of Psychology, Laval University, 2325 Rue des Bibliothèques, Quebec City, QC G1V 0A6, Canada; lily.bellehumeur-bechamp.1@ulaval.ca (L.B.-B.); maxime.legendre.1@ulaval.ca (M.L.); 2 Centre d’Expertise Poids, Image et Alimentation (CEPIA), Institute of Nutrition and Functional Foods, 2440 Bd Hochelaga, Quebec City, QC G1V 0A6, Canada; 3 Interdisciplinary Research Center on Intimate Partner Relationship Problems and Sexual Abuse (CRIPCAS), University of Montreal, C.P. 6128, Montreal, QC H3C 3J7, Canada

**Keywords:** binge eating, maltreatment, bullying, childhood interpersonal trauma, personality, emotional regulation, cognitive regulation, obesity

## Abstract

**Background/Objectives**: Binge eating (BE) is associated with physical and psychological consequences, such as obesity and reduced quality of life. The relationship between binge eating and childhood experiences of interpersonal trauma has been explored, yet few studies focus on the processes that may explain this association. In this regard, some personality traits and maladaptive cognitive-emotional regulation may help explain this relationship, as they have been associated, respectively, with BE and childhood interpersonal trauma. The purpose of this study is to explore the complex processes that link childhood interpersonal trauma and BE in a French-Canadian clinical adult population with obesity (BMI ≥ 30 kg/m^2^). **Methods:** This cross-sectional study included 148 participants aged 21 to 72, predominantly women of White ethnic background with a university degree, who were seeking treatment for eating or weight-related issues. They completed self-report questionnaires assessing maltreatment and bullying, BE, maladaptive cognitive-emotional regulation, and personality. Two multiple mediation models were tested to examine the indirect effects of personality and maladaptive regulation in the relationship between bullying and BE, as well as between maltreatment and BE. **Results**: The results revealed a significant indirect relationship between maltreatment and binge eating (BE), with personality traits and maladaptive cognitive-emotional regulation partially explaining this association. No direct effect was found for bullying, but a significant total indirect effect indicated that personality traits and maladaptive cognitive-emotional regulation play a mediating role in the relationship between bullying and BE. Finaly, self-directedness was found as a unique and significant contributor in both mediation models. **Conclusions**: This study draws attention to the multiple contributing factors in the relationship between interpersonal trauma and BE in adults with obesity. Further research is needed to gain a deeper understanding of the role of personality and maladaptive cognitive-emotional regulation in this relationship by focusing on individuals’ experiences.

## 1. Introduction

Binge Eating Disorder (BED) is characterized by recurrent episodes in which a large amount of food is consumed within a short period of time. It is accompanied by a feeling of loss of control, as well as significant distress regarding these episodes [1,2]. BED is the most prevalent eating disorder, with a global lifetime prevalence estimated at 1.9% [2]. Unlike other eating disorders, such as bulimia or anorexia, BED is not associated with compensatory behaviors like self-induced vomiting and excessive exercise. It is important to recognize that binge eating (BE) episodes can occur with varying frequency and intensity, which may not always meet the full diagnostic criteria for BED. In fact, BE episodes can occur at subthreshold levels, occurring less frequently or involving a quantity of food consumed that does not fulfill the criteria for a formal diagnosis. Nevertheless, individuals with BE can also experience significant psychological distress and physical consequences [3]. Beyond its psychological repercussions like reduced quality of life [2], BE is also significantly associated with an increased risk of obesity (body mass index; BMI ≥ 30 kg/m^2^) and the occurrence of medical conditions like diabetes [4,5]. According to the developmental framework of BED proposed by Tanofsky-Kraff and colleagues [6], loss of control eating is a key risk factor in BE onset, particularly when combined with other cognitive and psychological factors. Specifically, difficulties in executive functioning (e.g., impulsiveness, low self-regulation), heightened reward responsiveness (e.g., attentional bias toward food), and negative affectivity (e.g., emotional eating related to stress, anxiety, or interpersonal difficulties) are all important risk factors that can interact over time, contributing to the development of BE and BED. In this model, emotion regulation serves as a central mechanism that modulates the interaction between all key variables and contributes to the development of disordered eating behaviors.

Several studies have established a specific link between the emergence of BE episodes in adulthood and the experience of childhood interpersonal trauma [7,8,9]. Childhood interpersonal trauma is defined as experiences that compromise the safety or integrity of a child’s development [10,11]. Specifically, experiences of maltreatment, such as physical, psychological, or sexual abuse, as well as physical and psychological neglect, are common forms of childhood interpersonal trauma [8,12]. Some authors have emphasized the relevance of including bullying as a form of childhood interpersonal trauma because it represents a form of interpersonal violence particularly detrimental due to its chronicity [13]. Despite these findings, further research is needed to identify the underlying mechanisms through which childhood interpersonal trauma can be associated with BE episodes later in life [14,15,16]. According to some authors, emotional dysregulation may contribute to the explanation of this association [17,18].

Emotional dysregulation refers to an individual’s difficulty in effectively recognizing and managing negative emotions. This difficulty often stems from an inadequate development of emotional regulation, a skill that children typically acquire in a secure, supportive environment [17,18,19]. When this environment is disrupted by trauma or maltreatment, emotional regulation may fail to develop properly, leading to emotional dysregulation [20,21]. Emotional dysregulation is often identified as a consequence of childhood interpersonal trauma [22] and is central to the development and maintenance of BE because negative emotions are often a trigger of those episodes [23]. The developmental model of Tanofsky-Kraff and colleagues [6] reinforces these findings, positing that youth may use loss of control eating as a maladaptive emotional regulation strategy, aimed at avoiding or alleviating emotional discomfort or distress. Two studies have focused on emotional dysregulation as a mediating factor in the association between maltreatment and eating psychopathology among white female university students [24,25]. They both identified emotional dysregulation as a significant mediator in the association between emotional abuse and eating psychopathology using the Emotion Regulation Questionnaire (ERQ) [26] to assess emotional dysregulation. One of these studies, by Moulton and colleagues [24], also confirmed a significant mediating effect of emotional dysregulation between maltreatment (encompassing all forms of abuse and neglect) and eating psychopathology. Although the results confirmed the key role of emotional dysregulation, both studies relied on university student samples, which limits the generalizability of the findings when examining eating psychopathology. Additionally, while the ERQ provides a broad measure of emotional regulation skills, it would be beneficial to explore cognitive regulation strategies that might be used in response to trauma, as these often precede behavioral regulation strategies [26]. For instance, maladaptive cognitive-emotional regulation strategies, such as rumination and self-blame, are more prevalent in populations with BE behaviors [27]. Furthermore, cognitive strategies can play a critical role in how individuals process emotional experiences related to trauma, as they have been linked to the development of various psychological and behavioral difficulties, like depression, stress, and externalizing or internalized behaviors [28,29]. Focusing on the cognitive side of emotional regulation is pertinent for further understanding the mechanisms through which childhood interpersonal trauma can be associated with the occurrence of BE, while also offering new insights into an area that has not been explored in the existing literature. Moreover, the role of maladaptive cognitive-emotional regulation as a mediator in the context of bullying also remains underexplored in current research.

Personality is also a key factor frequently associated when exploring the relationship between childhood interpersonal trauma and BE [30,31,32,33]. In the context of the present study, we will use Cloninger’s bio-social framework of personality [34]. According to this framework, personality is defined by four temperament traits (i.e., harm avoidance, persistence, reward dependence, and novelty seeking), and three character traits (i.e., self-directedness, cooperativeness, and self-transcendence). Temperament refers to innate and stable aspects of personality, such as how individuals perceive their habits and abilities. In contrast, character reflects the individual’s ability to adapt and mature in response to life experiences and is more influenced by environmental factors. Cloninger’s model [34] is particularly relevant for this study, as it is one of the most widely used models in the study of eating disorders. Indeed, personality traits such as high harm avoidance and low self-directedness have been linked to binge eating (BE) in individuals with obesity [30,31,32,33]. Similarly, a strong connection exists between harm avoidance as well as lower levels of self-directedness and childhood interpersonal trauma [31]. While some authors emphasize the need for future research on the role of personality in understanding the processes potentially involved in the relationship between childhood interpersonal trauma and adult eating disorders [35,36,37], few studies have addressed it so far. To our knowledge, only one study examines the mediating role of personality in the relationship between childhood maltreatment and eating psychopathology (i.e., eating concern, weight concern) in a clinical adult population with different eating disorders [38]. Although the results failed to demonstrate the mediating role of personality, the authors highlighted the complexity and variability of the trauma-personality relationship as prior research already found a significant association between childhood trauma and specific personality traits, such as high harm avoidance and low self-directedness [31]. However, in this study, the fact that the authors chose to examine all personality traits simultaneously as mediators rather than focusing on specific traits consistently highlighted in the literature, may have significantly weakened the model’s power and reduced the likelihood of identifying meaningful associations. Furthermore, given that personality traits may vary according to belonging to one eating disorder rather than another [1,33], the decision to group all patients regardless of their eating disorder might also have contributed to the absence of relation found. Altogether, this highlights the need to refine the exploration of the factors that link childhood interpersonal trauma to the development of specific eating difficulties, such as binge eating, in adulthood.

Therefore, the present study aims to explore the complex processes that link childhood interpersonal trauma and BE in a clinical adult population with BE and obesity. Given the multifaceted nature of this relationship, the study will examine multiple mediators, including specific personality traits and maladaptive cognitive-emotional regulation, within a unified mediation model to better understand the underlying processes. Additionally, given the established links between cognitive-emotional regulation and certain personality traits [39,40], two distinct mediation models will examine their roles in the links between childhood maltreatment and BE, and between childhood bullying and BE, clarifying their unique and combined effects, while accounting for their mutual influences.

First, consistent with the existing literature, we hypothesize that there will be a significant and positive direct effect between childhood maltreatment and BE, as well as between bullying and BE. Second, we hypothesize that self-directedness, harm avoidance, and maladaptive cognitive-emotional regulation will significantly and partially explain the relationships between childhood maltreatment and BE, and between bullying and BE. Although no specific hypothesis was formulated regarding the unique contribution of each mediator when all variables are included simultaneously, each indirect pathway was explored to better understand the unique role of these mediators.

## 2. Materials and Methods

### 2.1. Participants

For this study, 148 adults with obesity seeking psychological help for eating or weight-related problems were recruited. To be included in the study, individuals needed to be (1) 18 years or older, (2) have a BMI ≥ 30 kg/m^2^ according to self-report height and weight, and (3) begin psychological counseling related to eating or weight difficulties. The sample was composed of 28 men and 118 women between 21 and 72 years old (M = 43.9 years old). The majority of participants had a White ethnic and cultural background (99.3%), with 58.9% having obtained a university degree and 51.3% earning an annual household income of over CAD 80,000. Mean BMI was 39.9 kg/m^2^, going from 30.0 up to 74.6 kg/m^2^.

### 2.2. Measures

#### 2.2.1. Binge Eating

The Binge Eating Scale (BES) [41] is a self-report scale measuring the presence and severity of BE. The questionnaire comprises a total of 16 items associated with BE, eight of which focus on behaviors and eight on cognitions and feelings. Each item is rated on a four-point scale from 0 to 3, leading to a continuous total score ranging from 0 to 46. In terms of interpretation, a total score of 17 or less indicates the absence of or few BE episodes; a score between 18 and 26 indicates the presence of moderate BE; and a total score of 27 or more indicates the presence of severe BE. The BES has been validated in French and showed strong psychometric properties, similar to the original version, including high internal consistency (α = 0.88) in clinical populations [42]. In this study, internal consistency was deemed satisfactory, exhibiting a Cronbach’s alpha coefficient of 0.86.

#### 2.2.2. Maltreatment

The Childhood Cumulative Trauma Questionnaire (CCTQ) [43] is a self-report questionnaire measuring five different types of maltreatment: physical abuse, sexual abuse, psychological abuse, psychological neglect, and physical neglect. This questionnaire was constructed with items based on prior research of childhood maltreatment [44,45,46,47]. Thirteen items are used to measure physical and psychological abuse, as well as physical and psychological neglect. Each item is measured using a Likert-type scale ranging from 0 (never) to 6 (every day or almost every day). In terms of sexual abuse, nine multiple-choice items are asked, focusing more specifically on the relationship with the abuser, the type of sexual act, and the frequency of abuse. In order to interpret the CCTQ, all types of childhood trauma are given a score of 0 (not experienced) or 1 (experienced). Once the rating has been completed, the scales can then be summed to obtain a total cumulative childhood trauma score. A Cronbach’s alpha coefficient of 0.88 was found in this study, indicating satisfactory internal consistency.

#### 2.2.3. Bullying

The Adolescent Peer Relations Instrument (APRI) [48] measures experiences of bullying during childhood and adolescence in three forms: physical, verbal, and social. The questionnaire consists of two 18-item sections, one dealing with bullying behaviors committed (Section A) and the other with bullying behaviors experienced (Section B). However, for the purposes of this study, only the section on bullying behaviors experienced (Section B) will be used. All items are measured using a Likert-type scale ranging from 1 (never) to 6 (every day), where a higher score represents a higher frequency of bullying. Internal consistency was high in this study, with a Cronbach’s alpha coefficient of 0.94.

#### 2.2.4. Harm Avoidance and Self-Directedness

The Temperament and Character Inventory (TCI-125) [49] is the abbreviated version (125 items) of the longer TCI scale (240 items). This self-report questionnaire is used to assess personality using seven measurement scales, including four for temperament (novelty seeking, harm avoidance, reward dependence, and persistence) and three for character (self-directedness, self-transcendence, and cooperation). Each scale consists of several true/false questions. The total score for each scale represents a mean score between 0 and 100. A higher scale score would indicate a stronger specific temperament or character. In this study, internal consistency was deemed adequate, showing Cronbach’s alpha coefficients ranging from 0.76 to 0.88.

#### 2.2.5. Maladaptive Cognitive-Emotional Regulation

The Cognitive Emotional Regulation Questionnaire (CERQ) [26] measures the conscious cognitive aspects involved in emotional regulation. This self-report questionnaire comprises 36 items assessing the frequency of use of various regulation strategies. Participants indicate their level of agreement on a 5-point Likert scale, ranging from 1 (almost never) to 5 (almost always). The instrument consists of nine subscales, grouped into two main categories of coping strategies in the face of adversity, maladaptive strategies (self-blame, blaming others, rumination, and catastrophizing) and adaptive strategies (perspective-taking, positive refocusing, positive reappraisal, acceptance, and planning refocusing). In this study, only the maladaptive strategies category, ranging from 0 to 20, will be used. Internal consistency was adequate, with a Cronbach’s alpha coefficient of 0.85.

### 2.3. Procedure

At their first session at the Centre d’Expertise Poids, Image et Alimentation (CEPIA), participants were offered by their therapist to complete an online battery of questionnaires on LimeSurvey to better identify their difficulties related to weight, body image, and eating. Informed consent was obtained from each participant before the questionnaires were administered. All participant data were also anonymized before being added to the database. This study was approved by The Laval University Research Ethics Committee (CERUL).

### 2.4. Statistical Analysis

All analyses were conducted using IBM SPSS 28 and Hayes’ Macro Process [50], with the involvement of a statistician in the process. Descriptive analysis and a Pearson correlation matrix were performed for the variables under study (BES, CCTQ, APRI, CERQ, TCI-125). Before testing the hypothesis, a multivariable outlier analysis was conducted on the database. Using the Mahalanobis distance to identify outliers, two participant’s data were excluded, as they deviated significantly from the overall pattern of the dataset. Thus, all analyses were performed with data from 146 participants in total. This decision was made to prevent potential anomalies that could lead to biased results. No other multivariate outliers were found for the dataset. Prior to conducting the multiple mediation analysis to test the two proposed hypotheses, the variables were standardized. Self-directedness, harm avoidance, and maladaptive regulation were all included simultaneously as mediators in the model to assess both direct and indirect effects. This also allowed for the examination of the unique mediation effect of each variable while controlling for the effects of the other two mediators. The indirect effect for each mediator was calculated by multiplying the effect of the independent variable (X, such as maltreatment or bullying) on each mediator (M, such as harm avoidance, self-directedness, and maladaptive cognitive-emotional regulation) by the effect of each mediator on the dependent variable (Y, such as BE). Specifically, the calculation involves three key paths: Path A represents the effect of X on M, Path B represents the effect of M on Y, and Path C is the total effect of X on Y. The total indirect effect is obtained by summing the individual indirect effects for each mediator in the model. The significance of all indirect effects was assessed using the bootstrap method, which involves resampling to estimate the precision of each effect. The results of these indirect effects are presented in the text.

## 3. Results

### 3.1. Descriptive Statistics and Correlations

Table 1 presents descriptive statistics of the variables under study. A significant association between BE and maltreatment was found, while no significant association with bullying was detected (Table 2). BE was also significantly negatively correlated with self-directedness, and positively correlated with harm avoidance and maladaptive cognitive-emotional regulation. Maltreatment and bullying were both significantly associated with the three studied mediators (self-directedness, harm avoidance, and maladaptive regulation). Finally, it is important to note that self-directedness, harm avoidance, and maladaptive cognitive-emotional regulation were all significantly and moderately correlated with each other.

### 3.2. Mediation Models

#### 3.2.1. Mediation Model 1

**Figure 1:**
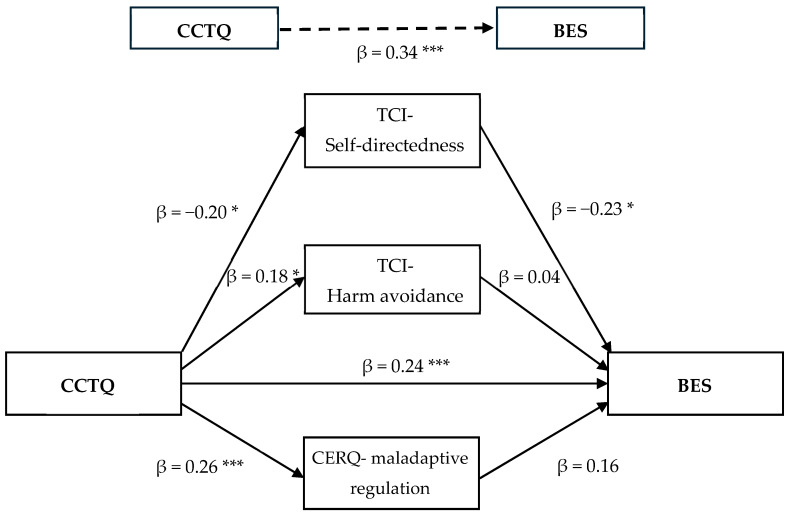
Mediation model of self-directedness, harm avoidance, and maladaptive cognitive-emotional regulation in the relationship between childhood maltreatment and binge eating in adults with obesity. * Significant correlations at *p* < 0.05. *** Significant correlations at *p* < 0.005. Note. The β value represents the standardized regression coefficient, controlled for the influence of other variables in the model.

The total indirect effect was significant β = 0.10, SE = 0.03, 95% CI = [0.04, 0.16], indicating that the combined effect of self-directedness, harm avoidance, and maladaptive cognitive-emotional regulation could significantly explain the process between childhood maltreatment and BE. Furthermore, the significant indirect effect of self-directedness β = 0.05, SE = 0.03, 95% CI = [0.00, 0.12], suggests that its unique contribution could partially explain the association between childhood maltreatment and BE in adulthood (Figure 1). Indirect effects also indicated that, when considered as single mediators, harm avoidance β = 0.01, SE = 0.02, 95% CI = [−0.03, 0.05] and maladaptive cognitive-emotional regulation β = 0.04, SE = 0.03, 95% CI = [−0.01, 0.10] were not significant, indicating that their single effect could not sufficiently explain the relationship between childhood maltreatment and BE. The direct effect of childhood maltreatment on BE was also significant (β = 0.24 *p* ≤ 0.005), indicative of a partial mediation. 

#### 3.2.2. Mediation Model 2

**Figure 2:**
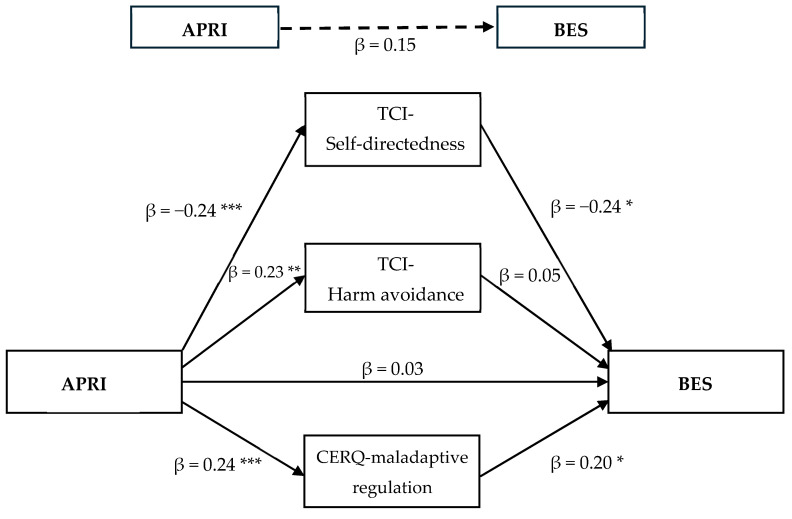
Mediation model of self-directedness, harm avoidance, and maladaptive cognitive-emotional regulation in the relationship between childhood bullying and binge eating in adults with obesity. * Significant correlations at *p* < 0.05. ** Significant correlations at *p* < 0.01. *** Significant correlations at *p* < 0.005. Note. The β value represents the standardized regression coefficient, controlled for the influence of other variables in the model.

The total indirect effect was significant, β = 0.12, SE = 0.04, 95% CI = [0.05, 0.19], indicating that the combined effect of harm avoidance, self-directedness, and maladaptive cognitive-emotional regulation could significantly explain the relationship between childhood bullying and BE in adulthood. Indirect effects of both maladaptive cognitive-emotional regulation β = 0.05, SE = 0.03, 95% CI = [0.00, 0.12] and self-directedness β = 0.06, SE = 0.03, 95% CI = [0.01, 0.13] were significant, indicating that their unique contribution could explain the process between childhood bullying and BE in adults (Figure 2). For harm avoidance, β = 0.01, SE = 0.02, 95% CI = [−0.03, 0.06], the indirect effect was not significant, meaning that it could not sufficiently explain the relationship between childhood bullying and BE. Because the direct effect of childhood bullying on BE was not significant (β = 0.03, *p* = 0.69), it is possible to conclude that there is a full mediation effect of the combined variables.

## 4. Discussion

This study explored the complex processes between childhood interpersonal trauma and BE in a clinical adult population with obesity. The findings, based on two mediational models that integrated multiple mediators and separately evaluated maltreatment and bullying in relation to BE, highlight the role of personality traits and maladaptive cognitive-emotional regulation strategies in these relationships. Several key findings can emerge from the results, confirming part of our hypothesis. First, the results indicated that experiences of maltreatment and bullying are associated with lower levels of self-directedness, higher harm avoidance, and maladaptive cognitive-emotional strategies. These results are consistent with those of deCarvalho and colleagues [31], who also found associations between these two personality traits and emotional abuse. The relationship found between childhood interpersonal trauma and maladaptive regulation was also supported by existing studies, where avoidance and emotional suppression were associated with maltreatment [51,52].

The presence of a direct effect between maltreatment and BE is aligned with our hypothesis and supported by previously mentioned studies on the subject [7,8,9]. However, the absence of a significant direct association between bullying and BE in adulthood is surprising given the well-documented link between these variables [53,54,55]. One potential explanation could be that bullying acts as a triggering factor, with its psychological consequences shaped by the individual’s personality and emotional regulation capacity, which could be understood through the vulnerability-stress model. Although bullying can be a significant stressor in a child’s life, the model posits that the child’s response to the event could be strongly influenced by certain vulnerability factors, including those associated with self-regulation and personality traits [56,57,58], thus supporting the indirect effect found.

Consistent with our hypothesis, a total indirect effect in both models was observed, through the combination of maladaptive cognitive-emotional regulation, harm avoidance, and self-directedness. This result suggests that the severity and the potentially wide range of impacts associated with maltreatment or bullying may overshadow the influence of unique factors in explaining the relationship under study. It thus highlights the importance of considering multiple factors simultaneously when exploring the processes involved in childhood interpersonal trauma—BE association in accordance with the multifactorial models of trauma’s impacts and the etiology of BE [6,7,52]. The indirect effects’ significance may explain the absence of a direct relationship between bullying and BE, contrary to what has been found in the literature [53,54,55]. The effect of bullying is indirectly manifested through the studied mediators, such as the frequent use of maladaptive cognitive-emotional strategies and maladaptive personality traits. While earlier studies have examined bullying as directly associated with BE, they also suggested that mediating factors need to be examined and integrated into a more complex and comprehensive model. Given all these findings, it is important to consider that while a total indirect effect was found in the present study, the processes explored may not fully capture this relationship. Thus, several other mediating factors in the relationship between maltreatment and BE have been documented in the literature, including insecure attachment, self-criticism, depression, anxiety, and dissociation [59,60]. Future prospective and qualitative studies could expand our understanding of these factors.

Among all unique contributors, the only one found systematically in both models was self-directedness. This finding is particularly interesting because, to our knowledge, no study has explored the mediational role of self-directedness in the association between both types of interpersonal trauma and BE in a clinical population with obesity. Specifically, it means that the difficulties to set goals, demonstrate responsibility and resourcefulness, as well as in accepting themselves as a person [34], may partially explain the process between the experience of a childhood interpersonal trauma and BE in adulthood. In relation to this finding, many studies have shown that self-directedness was negatively associated with BE as well as with childhood interpersonal trauma [31,61]. Van Riel and colleagues [62] also found that treatment-seeking individuals with BED and obesity exhibited lower levels of self-determination than individuals in the community with normal weight or with obesity. As conceptualized by Cloninger and colleagues [34,49], self-determination is a character that is shaped by life experience. Experiencing interpersonal trauma in childhood may impair a child’s sense of autonomy and self-control [63], potentially leading to externalized behaviors, such as binge eating, as an attempt to control their emotions.

The findings from this study reveal several implications for clinical psychology, notably by providing results that are generalizable to a clinical population experiencing BE. First, given the significant combined effect of self-directedness, harm avoidance, and maladaptive cognitive-emotional regulation on BE, clinicians should implement a comprehensive assessment that takes these factors into account alongside with the experience of childhood maltreatment. Furthermore, the chosen treatment, regardless of the therapeutic approach, should be tailored to meet the specific needs of individuals based on their unique combination of subjacent factors. Thus, a trauma-sensitive approach may be particularly relevant in this matter, as it focuses on the underlying elements of eating symptoms in relation to childhood interpersonal trauma [64]. In this context, incorporating strategies to help the patient regain a sense of control and autonomy in their life, by fostering self-reflection and addressing the avoidance and suppression of negative emotions, could promote the development of healthier coping mechanisms. Building tolerance for emotional discomfort and learning to effectively regulate emotions also emerge as key elements of the therapeutic process [65].

It is important to interpret the results in the light of their limitations. First, the overrepresentation of women in the sample reflects the typical demographics seen in clinical populations. This gender imbalance limits the generalizability of the findings to men with BE and a history of childhood interpersonal trauma. Furthermore, the lack of diversity in terms of education, ethnicity, and socio-economic status also limits the broader applicability of the results. Therefore, it will be important to replicate these findings with a more balanced and diverse sample, including individuals from various backgrounds. Second, the cross-sectional design prevents the establishment of causal relationships. Although childhood interpersonal trauma is most likely to occur prior to BE [1], it is possible that these events may have co-occurred for some participants, and the lack of a longitudinal approach prevents controlling for time-based effects. To strengthen the conclusions, it would be relevant to study the relationship between interpersonal trauma and BE with a prospective design, which would offer stronger evidence of causal relationships. Moving forward, there is also a need to qualitatively explore the experiences of individuals who have experienced childhood interpersonal trauma and BE in adulthood. This would lead to a deeper understanding of the various processes and nuances involved in this complex relationship.

## 5. Conclusions

This study has identified various processes involved in the emergence of BE episodes following childhood experiences of interpersonal trauma among adults living with obesity. The findings showed a direct association between maltreatment and the onset of BE in adulthood, emphasizing the importance of considering childhood interpersonal traumas in the psychological assessment of individual with BE and obesity. Additionally, the unique mediating role of self-directedness underscores the need for clinicians to consider this specific trait in their approach, while encouraging autonomy and self-reflection, all of which are essential in helping treatment-seeking individuals with BE. Additionally, the results highlight the significance of considering the combined effects of multiple factors when studying the relationship between childhood interpersonal trauma and BE in adults. This suggests that small improvements in each of these factors could potentially lead to significant overall improvements in treating BE. These findings not only enhance clinical understanding of this population but also have the potential to contribute to the development of more targeted therapeutic and preventive interventions, aimed at fostering resilience and adaptive cognitive-emotional regulation strategies in response to adversity. Furthermore, this study lays a foundation for future research to further explore and deepen the understanding of the pathways between childhood interpersonal trauma and BE in adulthood, particularly through prospective study designs to establish causal relationships. Future research should also qualitatively examine the perspectives of those affected to gain a deeper understanding of their experiences and the underlying mechanisms involved between childhood interpersonal trauma and BE.

## Figures and Tables

**Table 1:** Descriptive statistics.

Variables	M	SD	N
1. BES (/46)	22.47	8.89	146
2. CCTQ (/89)	12.54	12.66	146
3. APRI (/126)	45.95	18.89	146
4. CERQ-maladaptive	39.45	9.14	146
regulation (/80)			
5. TCI-Self-directedness (/100)	61.95	19.34	146
6. TCI-Harm avoidance (/100)	62.95	25.56	146

Note. BES = Binge Eating Scale; CCTQ = Childhood Cumulative Trauma Questionnaire; APRI = Adolescent Peer Relations Instrument; CERQ = Cognitive Emotional Regulation Questionnaire; TCI = Temperament and Character Inventory.

**Table 2:** Correlation matrix.

Variables	1	2	2	4	5	6
1. BES	-					
2. CCTQ	0.34 **	-				
3. APRI	0.15	0.39 **	-			
4. CERQ-maladaptive	0.37 **	0.26 **	0.24 **	-		
regulation						
5. TCI-Self-directedness	−0.39 **	−0.20 *	−0.24 **	−0.57 **	-	
6. TCI-Harm avoidance	0.28 **	0.18 *	0.23 **	0.45 **	−0.55 **	-

* Significant correlations at *p* < 0.05. ** Significant correlations at *p* < 0.01. Note. BES = Binge Eating Scale; CCTQ = Childhood Cumulative Trauma Questionnaire; APRI = Adolescent Peer Relations Instrument; CERQ = Cognitive Emotional Regulation Questionnaire; TCI = Temperament and Character Inventory.

## Data Availability

The data supporting the conclusions of this article will be made available by the authors at reasonable request.
